# Changes in the cerebrospinal fluid circulatory system of the developing rat: quantitative volumetric analysis and effect on blood-CSF permeability interpretation

**DOI:** 10.1186/s12987-015-0001-2

**Published:** 2015-03-10

**Authors:** Jean-François Ghersi-Egea, Anaïd Babikian, Sandrine Blondel, Nathalie Strazielle

**Affiliations:** BIP Platform, Faculté de Médecine RTH Laennec, INSERM U 1028, CNRS UMR5292, Lyon Neuroscience Research Center, Rue Guillaume Paradin, Cedex 08, 69372 Lyon, France; Oncoflam Team, INSERM U1028, CNRS UMR5292, Lyon Neuroscience Research Center, Lyon, France; Brain-i, Lyon, France

**Keywords:** Ventricle, Subarachnoid space, Influx constant, Blood–brain barrier, Meninges, Claudin, Embryo, Postnatal development

## Abstract

**Background:**

The cerebrospinal fluid (CSF) circulatory system is involved in neuroimmune regulation, cerebral detoxification, and delivery of various endogenous and exogenous substances. In conjunction with the choroid plexuses, which form the main barrier site between blood and CSF, this fluid participates in controlling the environment of the developing brain. The lack of comprehensive data on developmental changes in CSF volume and distribution impairs our understanding of CSF contribution to brain development, and limits the interpretation of blood-CSF permeability data. To address these issues, we describe the evolution of the CSF circulatory system during the perinatal period and have quantified the volume of the different ventricular, cisternal and subarachnoid CSF compartments at three ages in developing rats.

**Methods:**

Immunohistofluorescence was used to visualize tight junctions in parenchymal and meningeal vessels, and in choroid plexus epithelium of 19-day fetal rats. A quantitative method based on serial sectioning of frozen head and surface measurements at the cutting plane was used to determine the volume of twenty different CSF compartments in rat brain on embryonic day 19 (E19), and postnatal days 2 (P2) and 9 (P9). Blood-CSF permeability constants for sucrose were established at P2 and P9, following CSF sampling from the cisterna magna.

**Results:**

Claudin-1 and claudin-5 immunohistofluorescence labeling illustrated the barrier phenotype acquired by all blood–brain and blood-CSF interfaces throughout the entire CNS in E19 rats. This should ensure that brain fluid composition is regulated and independent from plasma composition in developing brain. Analysis of the caudo-rostral profiles of CSF distribution and of the volume of twenty CSF compartments indicated that the CSF-to-cranial cavity volume ratio decreases from 30% at E19 to 10% at P9. CSF compartmentalization within the brain changes during this period, with a major decrease in CSF-to-brain volume ratio in the caudal half of the brain. Integrating CSF volume with the measurement of permeability constants, adds to our understanding of the apparent postnatal decrease in blood-CSF permeability to sucrose.

**Conclusion:**

Reference data on CSF compartment volumes throughout development are provided. Such data can be used to refine blood-CSF permeability constants in developing rats, and should help a better understanding of diffusion, bulk flow, and volume transmission in the developing brain.

**Electronic supplementary material:**

The online version of this article (doi:10.1186/s12987-015-0001-2) contains supplementary material, which is available to authorized users.

## Background

The cerebrospinal fluid represents 50% of the brain extracellular fluid in adult mammals including humans. Recent findings indicate that the CSF circulatory system is not a mere passive drainage system for the brain, but is involved in neuroimmune regulation [1-3], cerebral detoxification [4], and cerebral delivery of various endogenous or exogenous molecules [5-7]. CSF circulation is complex. In adult, the fluid moves fast within the ventricular system, and reaches the cisterna magna and lateral recesses of the fourth ventricle through the foramina of Magendie and Luschka. From there it slows down while flowing through the subarachnoid and cisternal spaces, which occurs in three main directions. The CSF circulates 1) in a caudo-rostral direction, either dorsally around the cerebellum and cortex, or centrally through the internal cisterns, which in rodents are mainly formed by the ambient and quadrigeminal cisterns located between the midbrain and hippocampi/cortices, 2) in a caudo-rostral direction and ventrally through the cerebellopontine, interpeduncular, optic tract, and laminae terminalis cisterns, and around the olfactory bulbs, and 3) caudally along the spinal cord. In places such as the velum interpositum, ventricular and cisternal spaces are separated from each other only by a thin membrane which allows direct exchanges of material between the two fluid compartments [8,9].

The CSF is secreted and protected from peripheral harmful molecules by the choroid plexuses which develop from the neuroepithelium to form the major site of the blood-CSF barrier. Choroid plexuses also have the capacity to secrete into CSF different bioactive substances including guidance molecules as well as growth and differentiation factors. Owing to the early fetal development of the choroidal tissue, the choroid plexus-CSF system is likely to fulfill essential functions for brain development [10-12]. The current lack of comprehensive data on developmental changes in CSF spaces still impairs our understanding of CSF contribution to brain development, and limits the interpretation of blood-CSF permeability data. To address the early role of the CP-CSF system we first examined whether tight junction proteins are present at all brain barriers including CSF-bathed meningeal and cisternal vessels in embryonic day 19 (E19) rat fetuses, a requirement for generating a brain fluid environment which is controlled and independent from plasma composition before birth. Using a quantitative method to measure the volumes of all CSF compartments in rats at E19 and postnatal day 2 (P2) and 9 (P9), we describe the evolution of the CSF circulatory system in the developing brain during the perinatal period. We then applied some of these volume data to refine the interpretation of blood-CSF permeability constants in developing rats.

## Methods

### Tissue collection

Animal care and procedures were conducted according to the guidelines approved by the French ethical committee (decree 87–848), and by the European Community (directive 86-609-EEC). Sprague–Dawley rats, either pregnant time-dated females, or females with their litter, were obtained from Janvier (Le Genest Saint Isle, France). All animals were kept under identical conditions in standard cages, with free access to food and tap water under a controlled environment (12 h day-light cycles). Timed-pregnant female rats were anesthetized with inhaled isoflurane (5%) and body temperature was maintained with a heated pad. E19 animals were removed one by one from the mother and frozen in 2-methylbutane cooled at −50°C, in a position that allowed serial sectioning of the brain. P2 and P9 animals were decapitated and the severed head immediately frozen in 2-methylbutane cooled at −50°C. Limited expansion of the brain at the time of freezing led occasionally to a partial crack in the cranial bones. These brains were discarded from the analysis. Animal heads were kept at −80°C until use for serial sectioning.

### Immunohistochemistry

Immunohistochemical analysis of claudins in E19 rat brains (n = 3) was performed as previously described [13], using anti-claudin-1 polyclonal rabbit antibody 51–9000 and anti-claudin-5 mouse monoclonal antibody 35–2500 (Invitrogen, Carlsbad, CA, USA). Primary antibodies were diluted and used overnight at 4°C at a final concentration of 0.625 μg/ml for claudin-1, and 2 μg/ml for claudin-5. Alexa 555®-conjugated goat anti-mouse antibody A-21424 and Alexa 488®-conjugated goat anti-rabbit antibody A-11034 (Invitrogen) were used at a final concentration of 2 μg/ml at room temperature for 1 hour. Diamidine-2-phenylindole-dihydrochloride (DAPI, 236276, Roche Diagnostics, Manheim, Germany) was used as a fluorescent nuclear stain (0.1 μg/ml in saline phosphate buffer for 10 minutes at room temperature). Immunofluorescence was viewed and analyzed using an Imager Z1 fluorescence microscope equipped with a MozaiX motorized module, a Z-stack apotome system and a Digital Camera (Zeiss, Jena, Germany). Images were acquired using the AxioVs40 V 4.8 software (Zeiss).

### Morphometric analysis

The whole head (n = 4 for E19 and P9, n = 5 for P2) with intact meninges and CSF spaces was cut into 35-μm-thick serial sections using a CM 3050S cryostat (Leica, Nussloch, Germany). After every fifth section, a photograph of the remaining tissue block in the cryostat was taken with a sharp focus on the cutting plane using a VR-320 camera (Olympus, Tokyo, Japan). On these photographs, the tissue could be easily distinguished from the crystal-clear CSF present in the cisternal, subarachnoid, and ventricular spaces. Each photograph included a scale bar added in the cutting plane to enable quantification of the surface area of the cerebral structures and spaces of interest. In addition, every fifth section was collected on a glass slide and stained with hematoxylin and floxin. Micrographs of the sections were taken using a AxioCam ERc5s camera (Zeiss) connected to a SMZ800 stereotaxic microscope (Nikon, Amsterdam, Netherlands). The surface areas of the cranial cavity and of the CSF spaces of interest were delineated on the photographs and determined using the ZEN 2012 software (Zeiss). Micrographs of the stained sections were used to determine the surface area of the smaller intraparenchymal CSF-filled compartments (such as the third ventricle), when the borders were not sharp enough to be outlined in the tissue block photographs.

The total brain tissue volume and the volume of each fluid compartment were calculated by building the area under the surface area-distance curve, between the section where the given structure appears and the section where it disappears. Calculation was done using the composite trapezoidal rule for integration. CSF compartment volumes and tissue volumes were corrected by factors of 0.910 and 0.920, respectively, to account for water expansion at the time of freezing. The correction factor for tissue was adjusted for a water content in neonatal rat brain tissue of 88.5% [14,15]. The extremities of the brain cavity were defined as the appearance of the cerebellar subarachnoid space, and the disappearance of the olfactory bulbs.

### Protein measurement

Choroid plexuses from individual animals (n = 6 for both P2 and P9) were micro-dissected under a stereomicroscope and digested in 1 M sodium hydroxide. Protein concentration was determined by the method of Peterson [16] using bovine serum albumin as reference protein for the standard curve.

### Blood-CSF permeability measurement to [^14^C]-sucrose

Radio labelled [^14^C]-sucrose (435 mCi/mmol, Hartmann Analytic, Braunschweig, Germany) was administrated by intraperitoneal injection (12.5nCi/g) under slight gas anaesthesia, to prevent backflow. Blood was sampled from different animals of the same litter (n = 5 for P2 and n = 9 for P9) by cardiac puncture under pentobarbital anesthesia at times ranging from 3 to 30 minutes after injection. Additional animals from the litter were injected with 50nCi/g [^14^C]-sucrose. In these animals, blood was sampled twenty minutes after injection, rapidly followed by CSF sampling as follows. The skin was incised above the cistern magna, and a glass pipette was introduced in the cistern for CSF collection. The CSF was transferred in a microtube and the sample volume measured. Blood was collected in a heparinized tube and centrifuged at 5000 rpm for 5 min. Plasma and CSF samples were analyzed for radioactivity in a 1600 TR Packard scintillation counter, using Ultima-gold (Perkin-Elmer, Waltham, MA) as scintillation cocktail.

A litter-based area under the plasma [^14^C]-sucrose concentration-time curve (AUC) [17] was built from 0 to 30 minutes for each developmental stage using the composite trapezoidal rule for integration. Two permeability constants K_**in** csf_ and K_**w** csf_ were calculated as follows:

$$ {{\mathrm{K}}_{\mathbf{in}}}_{\mathrm{csf}}={\mathrm{C}}_{\mathrm{t}}/\mathrm{AU}{\mathrm{C}}_{0\to \mathrm{t}} $$

where C_t_ is the [^14^C]-sucrose concentration in CSF at the time of sampling t (ranging from 20 to 23.5 minutes), AUC _0→t_ the AUC recalculated from the litter-based experimental AUC and from the plasma concentration measured before CSF sampling. K_**in** csf_ is similar to an influx constant as defined previously for brain tissue.

$$ {{\mathrm{K}}_{\mathbf{w}}}_{\mathrm{csf}}={{\mathrm{K}}_{\mathbf{in}}}_{\mathrm{csf}}\times {\mathrm{V}}_{\mathrm{csf}} $$

where V_csf_ is the total volume of CSF in the brain. K_**w** csf_ represents the plasma volume cleared in the whole CSF of the animal.

## Results and discussion

**Figure 1:**
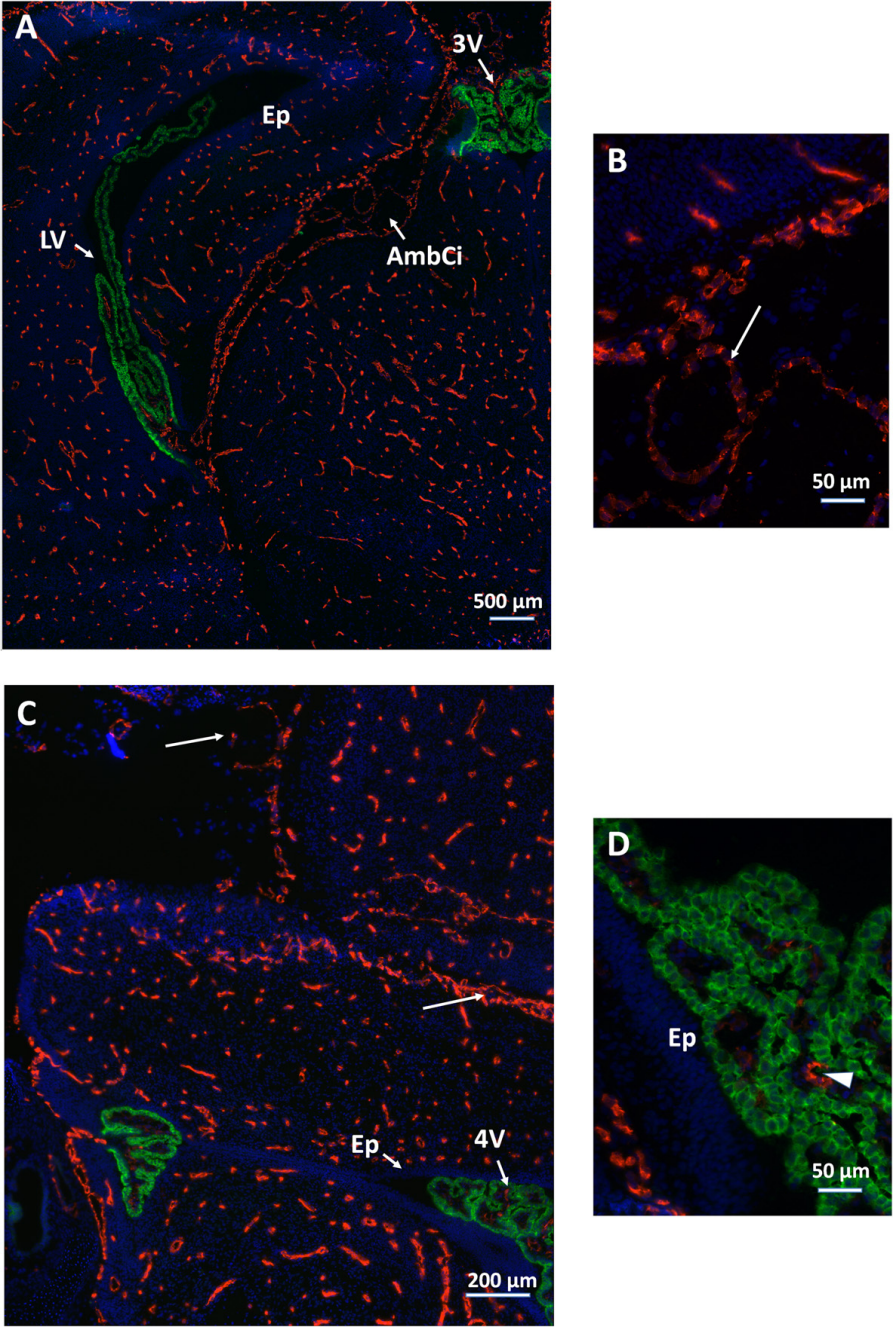
**Immunohistofluorescence analysis of claudin-1 and claudin-5 in E19 rat brain. A** and **B**: forebrain. The vascular network is labeled by claudin-5 antibodies (red), while both the third and lateral ventricles choroid plexuses are labeled by claudin-1 antibodies (green). **C** and **D**: Hindbrain. The cerebellar and medullar vascular networks are labeled by claudin-5 antibodies, while both the central and lateral parts of the fourth ventricle choroid plexus are labeled by claudin-1 antibodies. Note the intercellular junction labeling of the meningeal vessels (long arrows) such as that located in the ambient cistern (enlarged in **B**). Claudin-1 immunoreactivity at the periphery of choroidal epithelial cells is clearly seen in the enlarged fourth ventricle area **(D)**. Claudin-5 immunoreactivity is found in penetrating choroidal vessels (arrowhead), but not in the terminal vascular loops of choroidal villi. The ependyma (Ep) is devoid of staining. Other abbreviations: AmbCi: ambient cistern, LV, 3 V, 4 V: Lateral, third and fourth ventricles, respectively.

Antibodies against claudin-5, the hallmark constituent of tight junctions at the blood–brain barrier stained the entire microvascular network throughout the brain as well as endothelial junctions of meningeal vessels in E19 rat fetuses (Figure 1). The epithelial layer in all choroid plexuses was immunoreactive for claudin-1, the major protein of tight junctions at the blood-CSF barrier. This highlights that the barrier phenotype is acquired by blood–brain interfaces throughout the entire CNS in rat before birth. This stage can be compared to mid-gestation in humans. Staining of tight junction proteins in choroid plexus and meningeal vessels, but not in ependyma, infers that CSF-filled compartments are an integral part of the CNS at this developmental stage. In line with previous reports [11,13], it also suggests that the brain fluid composition is tightly regulated during development and is independent from plasma composition, a prerequisite for the CSF to fulfill its function in brain maturation. To better appreciate the extent of the CSF circulatory system and its implication in CNS physiology during perinatal development, we undertook a quantitative morphometric analysis of its compartments at E19, P2 and P9.

### Methodology of the quantitative morphometric analysis

**Figure 2:**
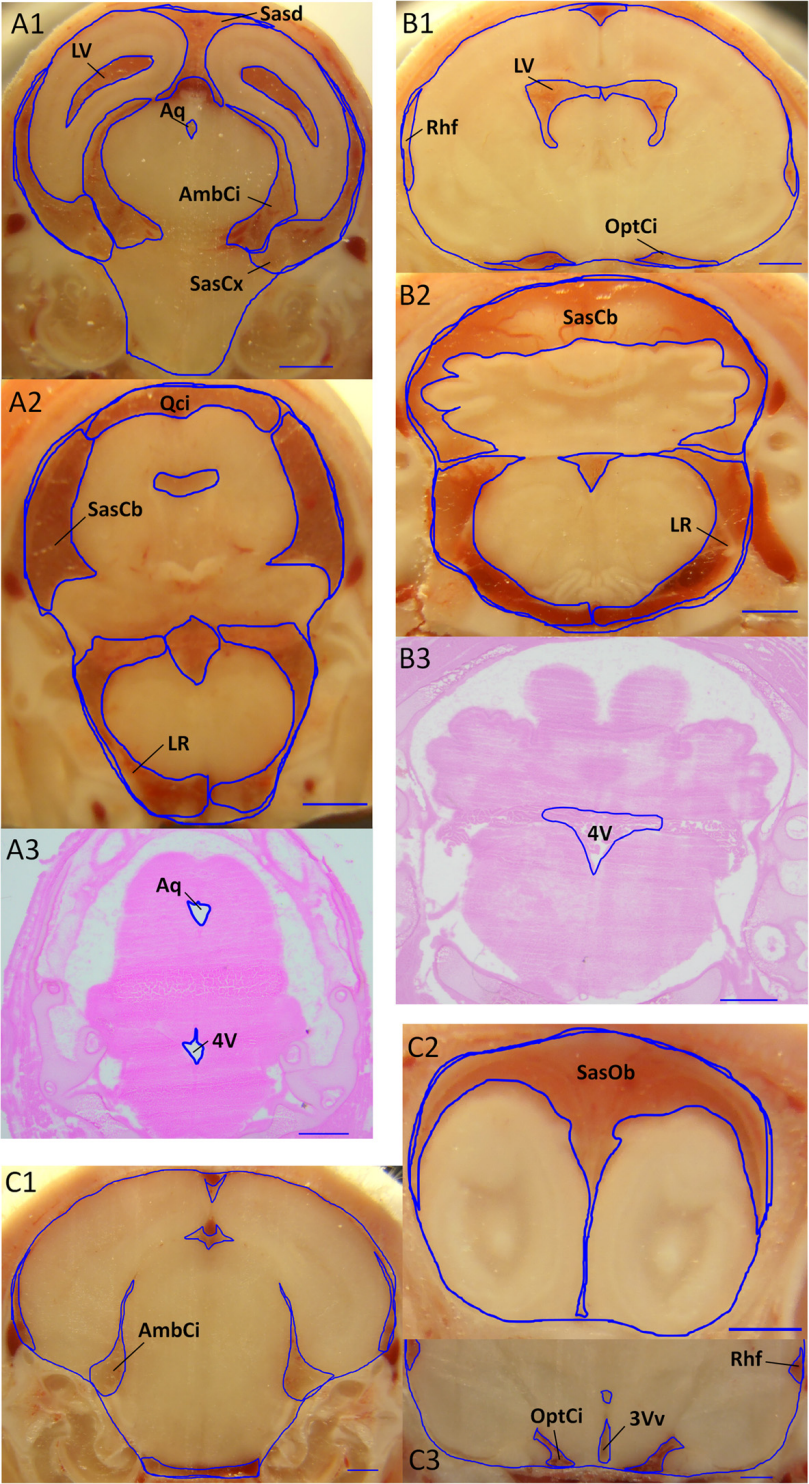
**Selected examples of cutting-plane photographs and histological sections used for morphometric analysis.** Images are from E19 **(A1-A3)**, P2 **(B1-B3)** P9 **(C1-C3)** animals. Fluid spaces and cranial cavity are delineated by a blue line. See Table 1 for abbreviations. Scale bar: 1 mm.

Brain ventricles of developing rats have previously been visualized by magnetic resonance imaging in animals suffering from hydrocephalus [18]. They were not observable in control animals at that time. Although the technique has since been adapted to small animal brain analysis, resolution remains a limiting factor to distinguish and to quantify the volumes of the various cisternal, subarachnoid, and ventricular compartments in normal developing rodents. We therefore conducted our morphometric study by a conventional approach based on the analysis of brain sections, with two distinct features. First, we generated these sections from whole frozen heads. Because of this unique aspect in the procedure, CSF is trapped within the subarachnoid, cisternal, and ventricular compartments. The shape and volumes of these spaces remain unchanged, with the exception of limited expansion of water at the time of freezing (Figure 2 and [8]). Second, photographs of the frozen tissue block taken at the cutting plane were preferred to micrographs of stained sections for surface area measurement, because on the latter the larger fluid compartments were easily distorted in the process of cutting and drying. Stained sections were used only for quantifying small fluid spaces when their boundaries could not be easily outlined on photographs. Examples of both cutting planes and stained sections covering a selection of CSF compartments from cerebellar to olfactory bulb subarachnoid spaces are shown in Figure 2. The size of CSF compartments changed between stages. To quantify these changes, we measured the total volume of the cranial cavity (from the cistern magna to the end of the olfactory bulbs), and the volume of twenty ventricular, cisternal, and subarachnoid compartments (listed in Table 1) at the three developmental stages. These two sets of data were used to calculate brain tissue volumes. All volumes were then corrected for water expansion at the time of freezing (see Method section). Brain tissue volume measured using this method was not statistically different from brain volume deduced from the weight of fresh brain sampled from age- and weight- matched animals, assuming an overall density of 1 (data not shown). This correlation provides a good index of measurement accuracy. The complete set of volume data obtained for the twenty compartments at the three developmental stages is displayed in a table see Additional file 1. It should be noted that with the exception of the ventricular system and lateral recesses of the fourth ventricle, all spaces of interest contain, in addition to CSF, arachnoid trabeculae, immune cells, and vessels traveling through the spaces before entering the brain. When appearing on sections, the major venous sinuses were excluded from the cisternal surface area measurements (e.g. Figure 2, A1 and C1). The CSF contained in the Virchow-Robin spaces which are located around penetrating vessels and are connected to the main subarachnoid and cisternal spaces could not be included, as the resolution of the method does not allow their visualization. Histological images of all the CSF spaces analyzed in this paper can be found in previous publications for the adult rat [3,8,19].

**Table 1:** **CSF compartments identified and analyzed in the study, and their corresponding abbreviations**

**SasC**	Subarachnoid spaces, caudal (E19 only)
**SasCb**	Subarachnoid spaces, cerebellum
**LR**	Lateral recesses of the fourth ventricle
**4V**	Fourth ventricle (central part)
**Aq**	Aqueduct
**Qci**	Quadrigeminal cistern
**Sasbl**	Subarachnoid spaces, base of the hindbrain
**AmbCi**	Ambient cistern
**SasCx**	Subarachnoid spaces, cerebral cortex
**LV**	Lateral ventricle
**MBCi**	Cistern of the mammiliary body
**3Vd**	Third ventricle, dorsal
**VI**	Velum interpositum
**OptCi**	Optic tract cistern
**3Vv**	Third ventricle, ventral
**Rhf**	Rhinal fissure
**Sasd**	Subarachnoid spaces, dorsal
**Cilt**	Cistern of the laminae terminalis
**Sasant**	Subarachnoid spaces, anterior
**SasOb**	Subarachnoid spaces, olfactory bulbs

The CSF compartments are listed in a caudo-rostral order.

### Developmental changes in CSF compartment volumes

**Figure 3:**
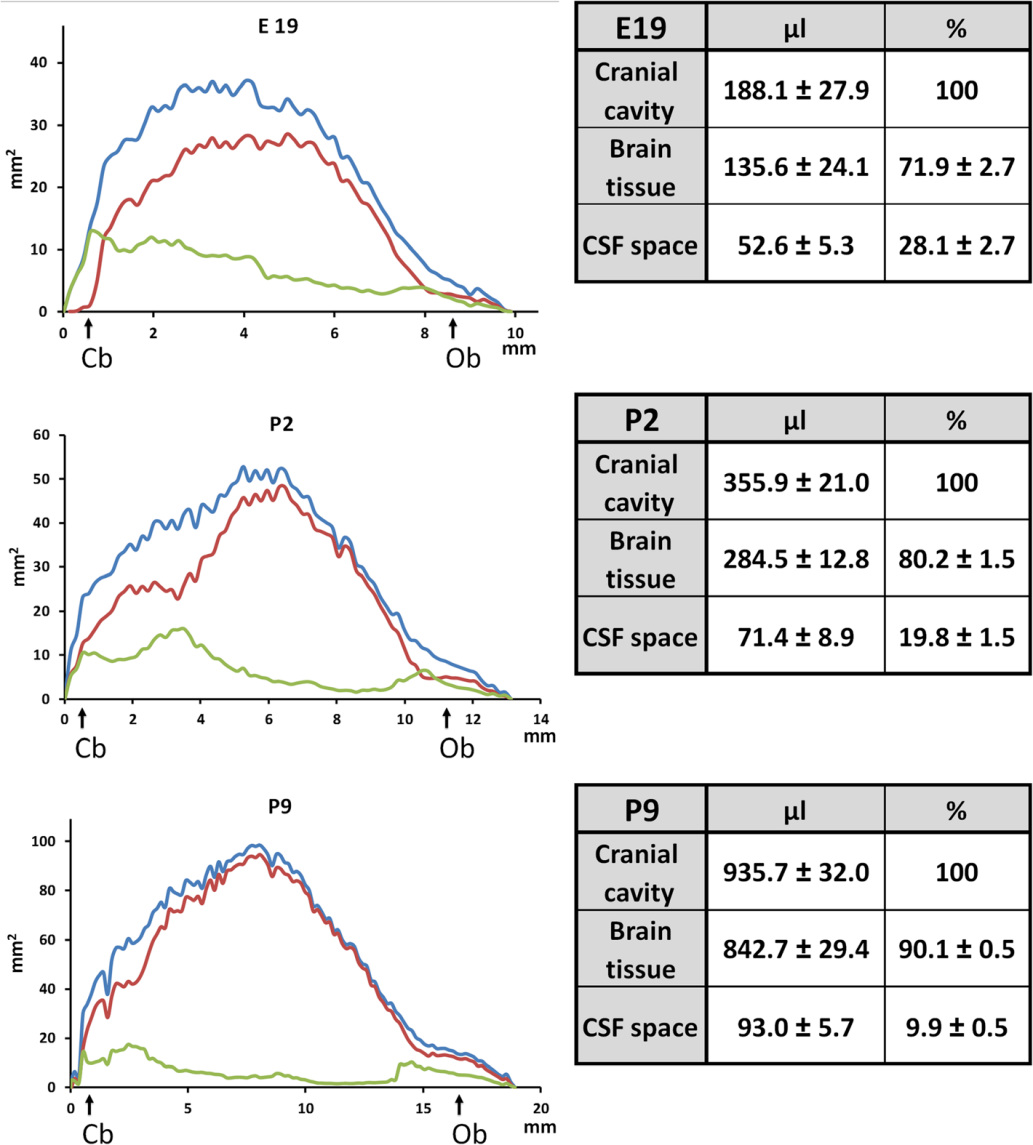
**Caudo-rostral profiles of brain tissue volume and CSF space in the developing rat brain. Upper panel**: 19-day-old embryo (E19), **middle panel**: 2-day-old rat (P2), **lower panel**: 9-day old rat (P9). Graphs show typical profiles combined from at least 3 animals. Positions of the cerebellum (Cb) and olfactory bulb (Ob) are indicated. Cranial cavity, brain tissue and CSF space profiles are represented in blue, red and green, respectively. Note scale difference between stages. Volumes corresponding to the three spaces are reported in the apposed tables as means ± SD, n = 4 (E19, P9), and n = 5 (P2).

The caudo-rostral profiles of the cranial cavity, brain tissue, and overall CSF spaces are shown for all three developmental stages in Figure 3. The volume values generated for these three parameters are listed in the adjacent tables. While the cranial cavity increased 1.9- and 5 times from E19 to P2 and P9, respectively, the total CSF volume increased only moderately over the same period. This translates into a decrease of the CSF-to-cranial cavity volume ratio from 28% at E19 to 20% at P2 and 10% at P9. The profiles show that CSF compartmentalization changes during development. There is a substantial decrease in the CSF space compared to the cranial cavity, which is mostly notable in the caudal half of the brain. We subdivided the CSF spaces in 7 main compartments to appreciate more precisely the geographic distribution of CSF over the developmental period (Table 2). Data expressed as a percentage of cranial cavity (left columns) indicated that the cerebellar subarachnoid spaces, ventricles, internal cisterns, and caudal part of the cortical subarachnoid spaces contribute most of the developmental decrease in CSF-to-cranial cavity volume ratio. Results expressed as a percentage of total CSF (right columns) show how the fluid distributes at each developmental stage. CSF distribution among the seven compartments differed more between E19 and P2 than between P2 and P9. A large part of the CSF was found in the internal cisterns at all stages. The cerebellar subarachnoid spaces and the caudal part of cortical subarachnoid spaces were also major CSF compartment at E19. The remote forebrain subarachnoid spaces and the hindbrain spaces mostly formed by the lateral recesses of the fourth ventricle became prominent in P9 animals. This resulted from the decrease in volume of other CSF compartments (relative to the cranial cavity volume). The lateral recesses of the fourth ventricle formed relatively large CSF spaces. They are generally not observable when the brain is sampled by conventional procedure, but were easily visualized by the method used in this study. These spaces are transitional areas between the ventricular and subarachnoid/cisternal system, in that no trabeculae or vessels pass through, and the border between the CSF and neuropil is not ependyma but the glia limitans. They extend dorsally along the medulla to become the spinal cord subarachnoid space. The latter also was relatively large in developing animals. In the anterior part of the spinal cord it ranged on average from 40% (E19) to 30% (P9) of the spinal canal volume (data not shown).

**Table 2:** **CSF compartmentalization in the different fluid-filled spaces of the developing brain**

		**% of cranial cavity**			**% of total CSF**		
	**Combined compartments**	**E19**	**P2**	**P9**	**E19**	**P2**	**P9**
Hindbrain spaces	LR, Sasbl	2.42 ± 0.13	2.92 ± 0.32	1.83 ± 0.35	8.7 ± 0.5	14.6 ± 1.6	18.4 ± 3.5
Cerebellar subarachnoid spaces	SasCb, SasC	4.70 ± 0.51	2.51 ± 0.59	1.38 ± 0.29	16.8 ± 1.8	12.5 ± 2.9	13.9 ± 3.0
Ventricles	LV, 3Vv, 3Vd, Aq, 4V	3.12 ± 0.68	1.48 ± 0.26	0.52 ± 0.05	11.2 ± 2.4	7.4 ± 1.3	5.3 ± 0.5
Internal cisterns	Qci, AmbCi, VI	6.02 ± 0.61	4.93 ± 0.44	1.96 ± 0.20	21.5 ± 2.2	24.6 ± 2.2	19.7 ± 2.0
Cortical subarachnoid spaces	SasCx, Rhf, Sasd	6.38 ± 0.68	3.23 ± 0.58	1.55 ± 0.12	22.8 ± 2.4	16.1 ± 2.9	15.6 ± 1.2
Basal cisterns of the midbrain	OptCi, MBCi	1.45 ± 0.23	1.78 ± 0.28	0.81 ± 0.15	5.24 ± 0.8	8.9 ± 1.4	8.1 ± 1.5
Forebrain subarachnoid spaces	Cilt, Sasant, SasOb	3.86 ± 0.95	3.28 ± 0.65	1.90 ± 0.24	13.8 ± 3.4	16.4 ± 3.2	19.1 ± 2.5

Data are mean +/− SD, n = 4 (E19, P9) or n = 5 (P2). See Table 1 for abbreviations.

The relative importance of hindbrain CSF spaces in E19 and their decrease throughout development is accounted for by the delayed development of the cerebellum, and the growth of the caudal part of cortex. Of note, cortical subarachnoid CSF is mainly found around this caudal part of the cortex and in the rhinal fissure, as the space between the surface of the cortex and the dura mater is narrow at all stages. This suggests that in the rodent, which does not display cortical circumvolution (but for the rhinal fissure), CSF flow is very limited between the pia-covered cerebral cortex and the arachnoid/dura mater. This is likely to be different in human, in which cortical foldings develop mainly between week 25 and 30 of gestation [20], resulting in changes in the pattern of flow at the cortical surface.

Finally the data show that the size of the ventricular system remains modest at all stages. The volume of the ventricles changed only moderately between E19 and P9, with a slight decrease for the lateral ventricles, an increase for the third ventricle and no change for the fourth ventricle (Additional file 1). By contrast, the size of choroid plexuses continues to enlarge during this period. The protein content of the lateral ventricle choroid plexuses increased from 100 ± 9 to 177 ± 13 μg between P2 and P9, and that of the fourth ventricle choroid plexus increased from 69 ± 9 to 131 ± 19 μg (mean ± SD of six animals at both ages). This increase in protein content parallels the change in size of dissected tissues observed under the stereomicroscope. This suggests there is increased flow of CSF through the ventricular system, which will be further enhanced by the concurrent increase in the rate of CSF secretion by the choroidal cells.

Overall, the volume data provided in this study for twenty compartments of the rat CSF system at different developmental stages (see Additional file 1) form reference figures which can be used in future studies to better delineate the role of the fluid environment in brain developmental processes. Taking into account the total CSF volume can also help to better interpret blood-CSF permeability measurements as exemplified below.

### Developmental changes in blood-CSF permeability constants for sucrose used as a marker of blood-CSF barrier efficiency

We measured [^14^C]-sucrose permeability constants by sampling CSF 20 min after injection. Blood concentration continuously increased during this period, minimizing the impact of tracer back flux. To account for changes in [^14^C]-sucrose plasma concentration over time we calculated the integrated plasma concentration-time product. We then generated influx constants K_**in***csf*_ with the assumption that the concentration measured in CSF drawn through the cisterna magna (5 to 10% of total CSF) is representative of the overall total CSF concentration. K_**in** csf_ values did not differ significantly between P2 and P9 animals (Table 3). A new CSF permeability constant K_**w** csf_ which takes into account the total volume of CSF in which the tracer is distributed (tables in Figure 3), was then calculated for each age (Table 3). K_**w** csf_ was also not statistically different between the two stages, but became significantly lower at P9 as compared to P2 when values were normalized for choroid plexus protein content (Table 3).

**Table 3:** **Blood-CSF permeability constants for [** ^**14**^ **C]-sucrose in the developing rat**

	**K**_**in csf**_	**K**_**w csf**_	**K**_**w csf**_ **/Pcp**
	**(10** ^**−3**^ **min** ^**−1**^ **)**	**(μl min** ^**−1**^ **)**	**(μl min** ^**−1**^ **mg** ^**−1**^ **)**
P2 rat	2.92 ± 0.54	0.21 ± 0.04	1.23 ± 0.23
P9 rat	2.61 ± 0.52	0.24 ± 0.05	0.79 ± 0.16**

Data are mean ± SD, n = 5 (P2) and n = 9 (P9). Pcp represents the protein content of all lateral and fourth ventricle choroid plexus. **Statistically different from P2 rat, two-tailed student t test for unequal variance, *p* < 0.01.

By using a short time point of 20 minutes, it is expected that a large proportion of the tracer reaches the CSF through the choroidal blood-CSF barrier and possibly across the meningeal vessel endothelium, another blood-CSF barrier site. The route across the blood–brain barrier proper is likely involved only to a limited extent. The presence of tight junctions that link the barrier cells of all choroidal epithelium, meningeal vessels, as well as parenchymal vessels ([11,13] and Figure 1) explains the limited blood-CSF permeability of [^14^C]-sucrose at both stages. The apparent permeability as assessed by K_**in** csf_ measurement remains however higher than for adult rat. In the latter, K_**in** csf_ was measured using a 30-minute time-point for inulin (a large molecule expected to diffuse 4 times less than sucrose across brain barriers), was 0.17 × 10^−3^ min^−1^ [21].

The constants K_w csf_ provide different information when comparing blood-CSF permeability at multiple developmental stages. K_**w** csf_ represents the virtual volume of plasma from which the tracer is cleared into the CSF per unit of time, which was about 0.2 μl.min^−1^ at both stages. Total cerebral blood flow across a 10-day-old rat brain, averaged from previous papers, is around 400 μl.min^−1^.g^−1^ tissue [22,23]. This is equivalent to 340 μl.min^−1^.brain^−1^ in P9 animals (brain volume taken from Figure 3). Therefore, only 0.06% of the tracer flowing through the brain vasculature reaches the CSF at P9. Data on cerebral blood flow in P2 animals and data on choroidal blood flow at both stages would have been useful to further analyze the data, but are not available to our knowledge. K_**w** csf_ measured in P9 animals becomes significantly lower than K_**w** csf_ measured in P2 animals when the values are normalized for choroid plexus protein content (Table 3). Assuming this protein content is proportional to the surface area of exchange across the blood-CSF barrier, this change in normalized K_**w** csf_ may be explained by a decrease in the paracellular permeability of the choroidal epithelium. The developmental expression profile of tight junction proteins, the similar claudin immunolocalization pattern in the choroid plexus at both ages [13], and restriction of a polar tracer by tight junctions observed by electron microscopy [11] suggest that junctions are already efficient at both ages. In addition, factors other than paracellular diffusion could also be involved in the apparent decrease in blood-CSF permeability between P2 and P9. A change in CSF turnover and an increase in CSF-to-tissue diffusion may affect CSF sucrose concentration differently at the two developmental stages. The capacity of the transcellular route for transport for plasma material across selected epithelial cells of the choroid plexus [24] may decrease during the postnatal period in rat. Hence, these factors, all independent from CSF volume, can possibly explain the higher CSF concentration of polar tracers measured at steady-state, several hours after injection, in P2 rats as compared to older animals [25]. By contrast, changes in the CSF distribution volume during the embryonic period appeared to have a substantial influence on apparent blood-to-CSF permeability. Thus, a 60% decrease in the CSF/plasma ratio for sucrose measured in conditions approaching steady-state in rat between E13 and E18 was attributed to the concomitant increase in CSF volume rather than changes in paracellular permeability of the choroidal epithelium [26]**.**

## Conclusion

The presence of tight junctions at blood–brain/CSF interfaces in 19-day rat embryos and the [^14^C]-sucrose permeability constants measured in 2- and 9-day-old rats confirm that the brain fluid environment is controlled and independent from plasma composition during development. Volumes of the different CSF fluid compartments measured during pre- and postnatal development in rat indicate not only a decrease in CSF-to-brain volume ratio, but also a geographical redistribution of the fluid especially around birth in rat. The volume data can be used to refine blood-CSF permeability constants in developing animals. The description of the different ventricular, cisternal and subarachnoid spaces and their respective developmental volume profile compared to total brain volume can be used to better understand cerebral fluid dynamic and diffusional/bulk flow movement of solutes in the developing brain. These fluid spaces are involved in volume transmission, clearance of potentially deleterious molecules, and immune cell migration within the brain These results will enable a better appreciation of the role of fluid compartments in brain development, neuroimmune surveillance of the infant, and in neonatal injuries.

## Additional file

Additional file 1:
**Volumes of the CSF compartments in 19-day-old rat embryos (E19), 2-day old rats (P2), and nine-day old rats (P9).** Mean and standard deviations for the volumes of all the twenty compartments measured as listed in Table 1.

## Abbreviations

AUC: Area under the curve
CSF: Cerebrospinal fluid
CNS: Central nervous system, anatomical abbreviations: see Table 1
