# Supplementary material for: Changes in the cerebrospinal fluid circulatory system of the developing rat: quantitative volumetric analysis and effect on blood-CSF permeability interpretation
Source: Fluids Barriers CNS. 2015 Mar 10;12:8. doi: 10.1186/s12987-015-0001-2 (PMC4365764; doi:10.1186/s12987-015-0001-2)
Supplement: Additional file 1: — Volumes of the CSF compartments in 19-day-old rat embryos (E19), 2-day old rats (P2), and nine-day old rats (P9). Mean and standard deviations for the volumes of all the twenty compartments measured as listed in Table 1. [file 12987_2015_1_MOESM1_ESM.pdf]

<u>Additional file 1</u>: Volumes of CSF compartments in 19-day-old rat embryo (E19), 2-day-old rats (P2) and 9-day-old rats (P9)

	E19		P2		P9	
	Mean	SD	Mean	SD	Mean	SD
Animal weight (g)	nd	nd	7.95	0.30	18.13	0.22
SasC	5.12	1.10				
SasCb	3.72	0.42	8.93	2.09	12.88	2.76
LR	3.68	0.41	5.58	0.93	13.85	2.38
4V	0.81	0.09	0.98	0.16	0.81	0.25
Aq	1.17	0.14	0.73	0.14	0.26	0.07
Qci	3.79	0.77	6.93	1.57	8.61	0.73
Sasbl	0.87	0.44	4.81	0.46	3.23	1.16
AmbCi	6.98	0.44	9.87	0.88	8.16	1.97
SasCx	10.16	1.22	7.27	1.76	7.17	1.40
LV	3.34	0.95	2.95	0.74	1.89	0.21
MBCi	0.44	0.20	2.76	0.83	2.64	0.36
3Vd	0.35	0.06	0.44	0.08	1.41	0.29
VI	0.55	0.11	0.76	0.31	1.56	0.17
OptCi	2.29	0.39	3.57	0.21	4.92	1.09
3Vv	0.20	0.21	0.15	0.03	0.52	0.13
Rhf	-	-	2.43	0.44	2.80	0.34
Sasd	1.85	0.30	1.80	0.35	4.50	0.18
Cilt	0.61	0.43	0.64	0.25	1.35	0.48
Sasant	3.49	1.18	2.57	0.86	4.26	1.31
SasOb	3.16	0.44	8.48	1.34	12.15	1.44

Mean and Standard deviation (SD) from 4 (E19, P9) and 5 (P2) animals are given in µl. See Table 1 in the text for CSF compartment abbreviations.

nd: not determined. -: not visible.
